# Extreme temperatures in Southeast Asia caused by El Niño and worsened by global warming

**DOI:** 10.1038/ncomms15531

**Published:** 2017-06-06

**Authors:** Kaustubh Thirumalai, Pedro N. DiNezio, Yuko Okumura, Clara Deser

**Affiliations:** 1 Institute for Geophysics, Jackson School of Geosciences, University of Texas at Austin, J. J. Pickle Research Campus, Building 196, 10100 Burnet Road (R2200), Austin, Texas 78758, USA; 2 Climate Analysis Section, Climate and Global Dynamics Division, National Center for Atmospheric Research, Boulder, Colorado 80305, USA

## Abstract

In April 2016, southeast Asia experienced surface air temperatures (SATs) that surpassed national records, exacerbated energy consumption, disrupted agriculture and caused severe human discomfort. Here we show using observations and an ensemble of global warming simulations the combined impact of the El Niño/Southern Oscillation (ENSO) phenomenon and long-term warming on regional SAT extremes. We find a robust relationship between ENSO and southeast Asian SATs wherein virtually all April extremes occur during El Niño years. We then quantify the relative contributions of long-term warming and the 2015–16 El Niño to the extreme April 2016 SATs. The results indicate that global warming increases the likelihood of record-breaking April extremes where we estimate that 29% of the 2016 anomaly was caused by warming and 49% by El Niño. These post-Niño Aprils can potentially be anticipated a few months in advance, and thus, help societies prepare for the projected continued increases in extremes.

## Abstract (web summary)

Record temperatures in mainland Southeast Asia in April 2016 had severe impacts on the population. Thirumalai *et al*. show that all April extremes occur after El Niño years, and that global warming has increased the likelihood of such extremes.

Mainland southeast Asia (MSA) encountered its warmest monthly mean surface air temperatures (SATs) in April 2016 since record-keeping began in the mid-twentieth century^1^,^2^,^3^. Apart from surpassing national temperature records in MSA, this event disrupted crop production, imposed societal distress and resulted in peak energy consumption^4^,^5^,^6^. Investigating the causes of such an event is essential to anticipating future extremes, especially in light of ongoing and projected long-term warming in the region^7^.

While it is unmistakable that the Earth is warming globally due to the effect of increasing greenhouse gases^8^, the impact of warming at spatial scales of MSA, including the causes of extremes such as the April 2016 event, is more uncertain^9^. In the MSA region, which encompasses Cambodia, Laos, Myanmar, Thailand, Vietnam and peninsular Malaysia (Fig. 1a), April is particularly prone to experiencing record-breaking SATs as it is climatologically the warmest month of the year (Fig. 1b).

Studies using climate models suggest that tropical land areas, particularly Central Africa, the Maritime Continent and the Indian Ocean rim, will show the most rapid and robust intensification of peak seasonal temperatures, with at least 60% of regional land areas exceeding the late twentieth century maximum around the mid-twenty-first century^10^,^11^,^12^,^13^. Observations show that large parts of the Earth have experienced a significant local shift towards warmer temperatures in the summer season, particularly at lower latitudes^11^,^12^,^13^,^14^, with one recent study^15^ indicating that MSA has likely experienced the emergence of distinctly higher peak seasonal SATs in response to global warming since the year 2000. Perhaps the hot April of 2016 is an indication that the MSA region is already experiencing a departure from its pre-industrial climate, as predicted by the models? These studies indicate that on the spatial scales of MSA, long-term warming is expected to emerge sooner in the lower latitudes relative to anywhere else on the planet. This occurs because lower latitudes experience naturally lower year-to-year variability compared to higher latitude continental regions, thus allowing for an earlier detection of climate departures from a reference state^12^,^16^,^17^. For this reason the Intergovernmental Panel on Climate Change 5th Assessment Report concluded with high confidence that relative to natural variability, near-term increases in seasonal and annual mean temperatures are expected to be larger in the tropics and subtropics than in mid-latitude regions^8^.

In this work, we use observations as well as an ensemble of global warming simulations to understand the relationship between long-term warming and natural climate variability in MSA, and their combined impact on extreme SATs. We demonstrate that extreme April SATs in the region occur after the peak of El Niño years and characterize the impact of long-term warming on these events. Finally, we quantify the relative contributions of the El Niño/Southern Oscillation (ENSO) and long-term warming in producing the April 2016 event and demonstrate that with the continued march of ongoing global warming, record-breaking SATs in MSA will occur more frequently in the future.

## Results

### El Niño and April SATs in MSA

**Figure 1:**
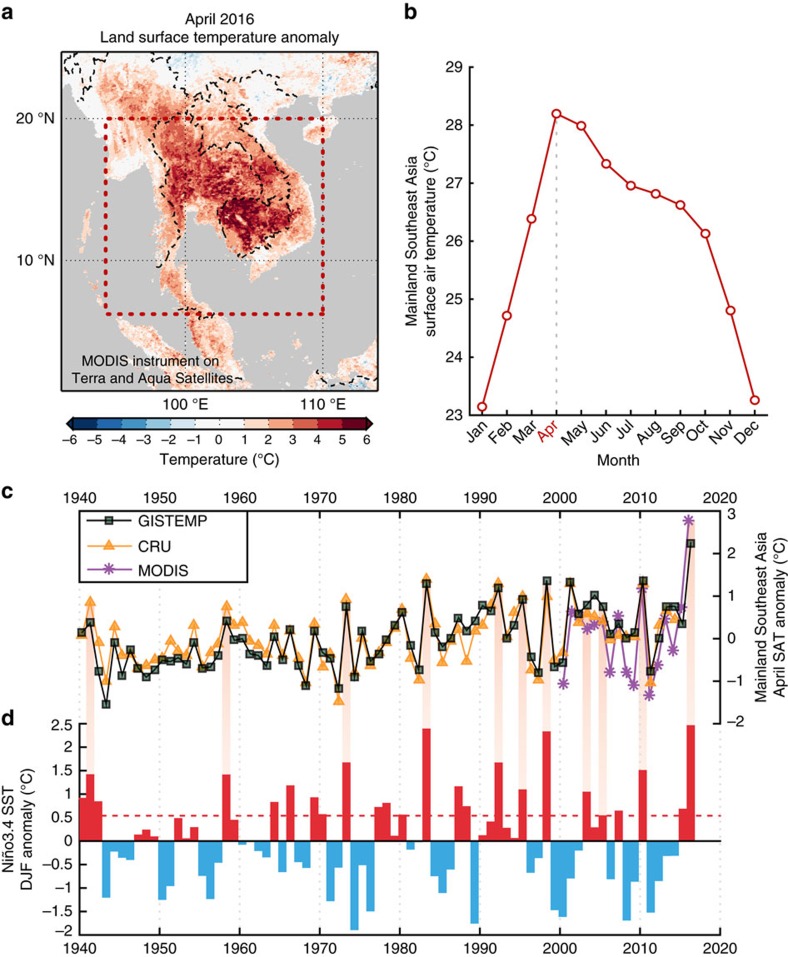
Surface temperatures over Mainland Southeast Asia. (**a**) Satellite-derived (MODIS instrument on Terra and Aqua satellites) land surface temperature anomaly during April 2016 in the Mainland Southeast Asia (MSA) region (base period: 2000–2016), where the dashed red box represents the region selected for our analysis. (**b**) Surface air temperature (SAT) climatology in MSA based on the entire CRU data set (1901–2014), which indicates that April is the warmest month in the region. (**c**) April SAT anomalies in the MSA region from the GISTEMP (green squares) and CRU data sets (yellow triangles) spanning from 1940 to present (base period: 1951–1980; ref. 34). MODIS-based land surface temperature anomalies for MSA (purple stars) are also plotted with the reference base period adjusted to equal the April average of the other two data sets (**d**) The December–January–February (DJF) anomaly of sea-surface temperatures (SST) in the Niño-3.4 region, Central Pacific Ocean, where SSTs are taken from the HadISST1.1 data set. As an indicator of El Niño events, a dashed red line is plotted at 0.5 °C. Shaded red bars connect El Niño events to hot Aprils in MSA.

Climate in the MSA region exhibits pronounced year-to-year variability that is strongly linked to the ENSO phenomenon^7^,^18^. April SATs over MSA are highly correlated (*r*=0.73) with the Niño-3.4 sea-surface temperature (SST) index, a common metric used to monitor ENSO variability^19^ that peaks during the December–January–February (DJF) season (Fig. 1). Positive DJF Niño-3.4 SST anomalies are associated with El Niño events and correspond to positive April SAT anomalies in MSA (Fig. 1d; hereafter ‘post-Niño Aprils'; see also Supplementary Fig. 2). While studies have suggested that this link with ENSO can be exploited to anticipate SAT anomalies after peak SSTs during the DJF season^14^,^18^,^20^, its impact on extremes in MSA during April remains poorly understand, especially in light of ongoing long-term warming^9^.

**Figure 2:**
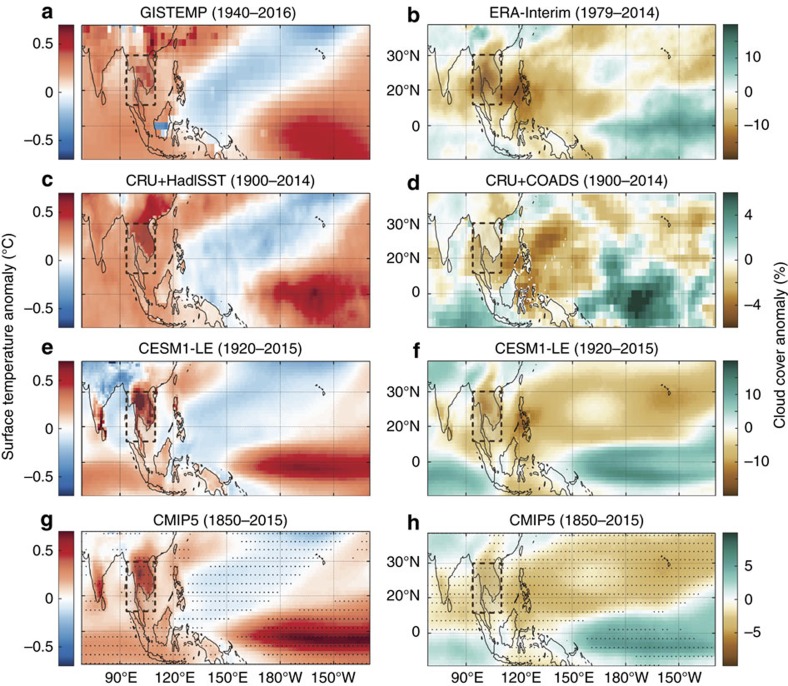
Post-Niño April composites. Composites of (**a**–**d**) observed and (**e**–**h**) simulated (left) SAT and (right) cloud cover anomalies during April after El Niño events. The SAT observations are from the (**a**) GISTEMP datas et (1940–2016) and (**c**) CRU and HadISST1.1 data sets (1900–2014) whereas the cloud cover observations are from the (**b**) ERA-interim reanalysis data set and (**d**) the CRU and ICOADS data sets. The simulated composites are from (**c**) the CESM1-LE model with data from 1920 to 2015 (ref. 22) and the CMIP5 Ensemble from 1850 to 2015 (ref. 23; stippling indicates agreement between majority of the ensemble members). Composites were produced by averaging April anomalies after the peak of El Niño events where the simulated composites are the average composite among the 39 CESM1-LE members. El Niño events were identified as those years with peaks of the Niño-3.4 SST index larger than 0.5 standard deviations in each (observational and simulated) data set. The CESM1 composite SAT anomalies are scaled down by a factor of 0.45 over both land and ocean so that the April composite SST anomalies averaged over the Niño-3.4 region agree in magnitude with the observed April HadISST1.1 composite. No scaling was performed for the cloud cover data simulated by CESM nor for the SAT and cloud cover simulations from the CMIP5 ensemble.

We explored the MSA April-El-Niño link further through a composite analysis of observed SATs during post-Niño Aprils. SAT anomalies from the GISTEMP^2^ and CRU^3^ (land) and HadISST^21^ (oceans) data sets show that, on average, post-Niño Aprils are characterized by warming over MSA (Fig. 2a,c), with enhanced warming over land. The April temperature response in the region to El Niño is among the largest in the world. This pattern is robust across the observational data sets, which show that, on average, post-Niño Aprils are accompanied by 0.6–0.7 °C of warming in MSA (Fig. 2a,b). A similar pattern of land amplification of the post-Niño April heat is seen in an ensemble of simulations of twentieth and twenty-first century climate performed with the Community Earth System Model Version 1 Large Ensemble (CESM1-LE) (Fig. 2e) and the CMIP5 suite of model members (Fig. 2g), confirming the observed MSA April-El-Niño link^22^,^23^.

These composite analyses reveal that peak seasonal SATs in the MSA region are highly sensitive to El Niño. Previous studies proposed that changes in atmospheric circulation associated with El Niño reduce cloud cover over the land areas of the Maritime Continent and Indian Ocean rim causing the associated warming^20^,^24^,^25^,^26^. To test whether clouds play a role causing extremes SATs over MSA, we performed a composite analysis of cloud cover anomalies during post-Niño Aprils using land- and ship-based observations together with reanalysis data as well as CESM1-LE and CMIP5 output (Fig. 2). We find that cloud cover observations from the ERA-interim data set^27^ and CRU (land) and ICOADS (ocean) data sets^3^,^28^ show, on average, reduced cloud cover over MSA during post-Niño Aprils (Fig. 2b,d). These reductions in cloud cover appear to be linked with increased cloud cover over the central Pacific, suggesting that the SST anomalies associated with decaying El Niño events could be driving anomalous overturning circulation in the atmosphere and thereby creating drought conditions over southeast Asia (Supplementary Figs 2 and 3; ref. 20). Our analysis shows that during April, the area of reduced rainfall and cloud cover has moved from the Maritime Continent, where El Niño impacts are more pronounced at their winter peak, to over MSA and the Philippines, explaining the region's sensitivity to ENSO.

The CESM1-LE and CMIP5 ensemble means simulate remarkably realistic patterns of surface temperature (Fig. 2e,g) and cloud cover anomalies (Fig. 2f,h), compared with observations from different data sets. This lends further support for a robust relationship between El Niño and hot April extremes in MSA inferred from observations. The CESM1-LE also simulates ENSO realistically (DiNezio *et al*. in prep) including the tropical SST patterns as well as circulation anomalies and teleconnections (see Methods for details on these simulations). The realism of CESM1 allows us to use data from the Large Ensemble, consisting of output from 39 simulations of twentieth and twenty-first century climate^22^, to explore the effect of long-term anthropogenic warming as well as El Niño on extreme seasonal temperatures over MSA.

### Histogram analysis of April SATs

**Figure 3:**
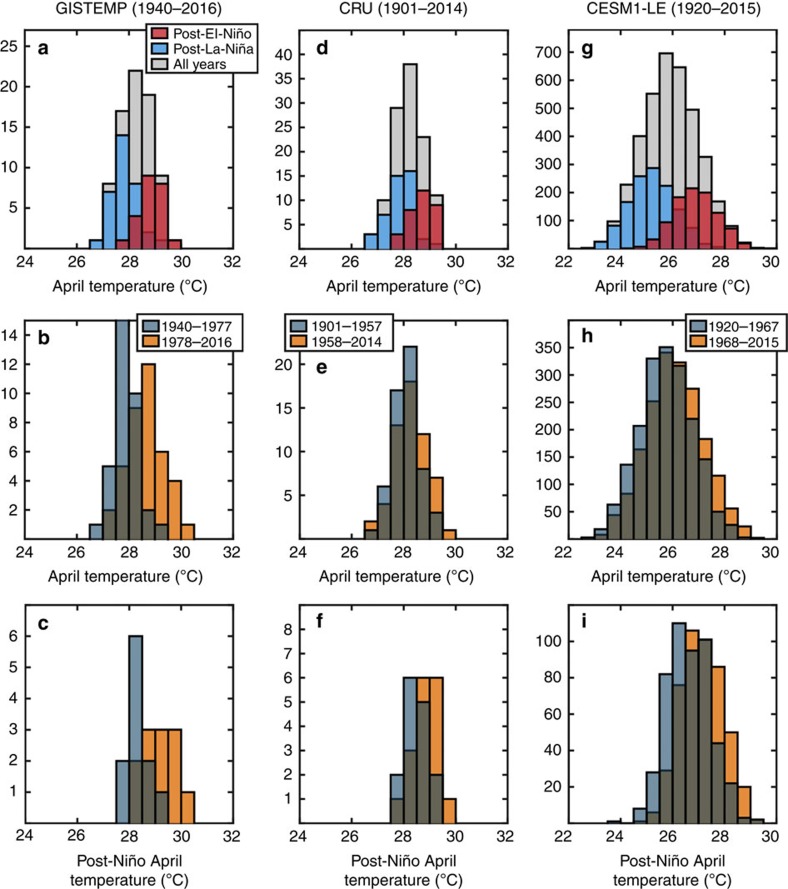
Histograms of April SATs. SATs for Mainland Southeast Asia (MSA) are taken from the GISTEMP (1940–2016; **a**–**c**) and CRU (1901–2014; **d**–**f**) observational data sets along with simulations from the CESM1-LE (1920–2015; **g**–**i**). Histograms in the top panel (**a**,**d**,**g**) delineate all the April data (grey) into those occurring after El Niño (red) and La Niña (blue) events over the entire time period. Histograms in the middle panel (**b**,**e**,**h**) separate the available April surface temperature data into two equal time periods (dark blue—earlier; orange—latter) over the length of the individual data sets. The bottom panel histograms isolate post-Niño-April events in the observations (**c**,**f**) and simulations (**i**) over the time period indicated in the middle panel, i.e., they separate the post-Niño events (red) in the topmost panel into the subperiods indicated by the middle panel for each of the data sets. All data sets were linearly detrended prior to analyses where the top panel histograms (**a**,**d**,**g**) were detrended over the entire time period for each data set, whereas linear detrending was performed for each subdivided time period in the subsequent analysis. We found that over their respective time periods for both GISTEMP (1940–1977; 1978–2016) and CRU (1901–1958; 1959–2014) data sets, the mean was significantly larger in the more recent subperiod (*t*-test: *H*=*H*_a_; *P*<0.01) whereas the variance was not significantly different between the subperiods (*f*-test: *H*=*H*_o_; *P*>0.05).

Do all El Niño events lead to hotter Aprils over MSA? We address this question by analysing the effect of El Niño and La Niña (El Niño 's cooler counterpart) on the distribution of April SATs. In both observations and simulations, after linearly detrending the data sets over their entire length to remove the long-term warming trend, we divided all April monthly mean SATs into distributions of post-El Niño and post-La Niña Aprils. The resulting distributions show that indeed the tails of the April MSA SATs are linked to ENSO variability. Both observations and model data show that post-El Niño Aprils are on average hotter (Fig. 3a–c, red bars) than post-La Niña Aprils (Fig. 3a–c, blue bars). Moreover, virtually all hot Aprils occurs following El Niño events (80% for GISTEMP, ∼73% for CRU, and ∼88% for CESM1-LE post-Niño April extremes had >29 °C detrended SAT values). The fact that the simulations accurately capture this shift in the distribution is indicative that the effect of ENSO on extreme SATs in MSA is robust.

Next, we looked into the effect of long-term regional warming on the observed and simulated distributions of all April SATs in MSA. Here, we divided the data sets into two equal subperiods and detrended the data over each subperiod (GISTEMP: 1940–1977 and 1978–2016; CRU: 1901–1958 and 1959–2015; CESM1-LE: 1920–1968 and 1969–2015). This ensures that the mean warming between the individual time periods is retained for each data set despite differing time periods (see also Supplementary Figs 4 and 5). The long-term warming results in distributions of April SATs with significant positive shifts in the mean according to a two-sample *t*-test (*P*<0.01). The distribution of SATs shifts due to this change in the mean and results in an increase of extreme SATs in the latter subperiod.

Lastly, we performed a similar analysis of only post-Niño Aprils (Fig. 3c,f,i). We find that the latter subperiods show more extreme post-Niño April SATs because of a significant shift in the mean of the distribution (two-sample *t*-test; *P*<0.01) but statistically indistinguishable variance (f-test; *P*>0.05). Changes in the tails of the distribution caused by shifts in the mean, similar to the ones identified here, are the most straightforward way in which global warming can lead to increased frequency of extremes^16^,^29^. Although increased extremes can also arise from increased variability, we do not observe statistically distinguishable variance between the two subperiods across any of these distributions (*f*-test; *P*>0.05). Thus, the mean shift in post-Niño April SATs underpins long-term warming as the most likely cause of the increasing extremes in MSA.

### Attribution of extreme April SATs

Based on this assessment of the roles of El Niño and long-term warming in modulating April SATs, we built a statistical model to attribute the 2016 event and other April extremes in MSA. The model is a simple multiple regression in the form SAT_April_=*αt*+*βt*^2^+*γT*+*δ*, where April SATs in MSA (SAT_April_) are regressed upon linear and quadratic components of the regional long-term warming trend^18^,^30^ represented by time in years (*t* and *t*^2^, respectively), a term proportional to DJF Niño-3.4 SST Index (*T*) capturing variability correlated with ENSO and a residual term (*δ*) capturing the effect of other climate variability and weather. This analysis yielded a fit that has a high correlation coefficient (*r*=0.83) with April temperature observations from 1940 to 2016 (Supplementary Fig. 6), indicating that long-term warming and DJF ENSO amplitude explain much of the SAT variability during April in MSA. The unexplained residual between our regression fit and the observed anomaly (termed ‘other') appears to be related to mid-latitude weather over Asia or local weather (see Supplementary Discussion). However, as the statistical model explains a large component of April SATs, we used the regression model to attribute the relative contributions of long-term warming and ENSO in producing observed extremes in MSA.

**Figure 4:**
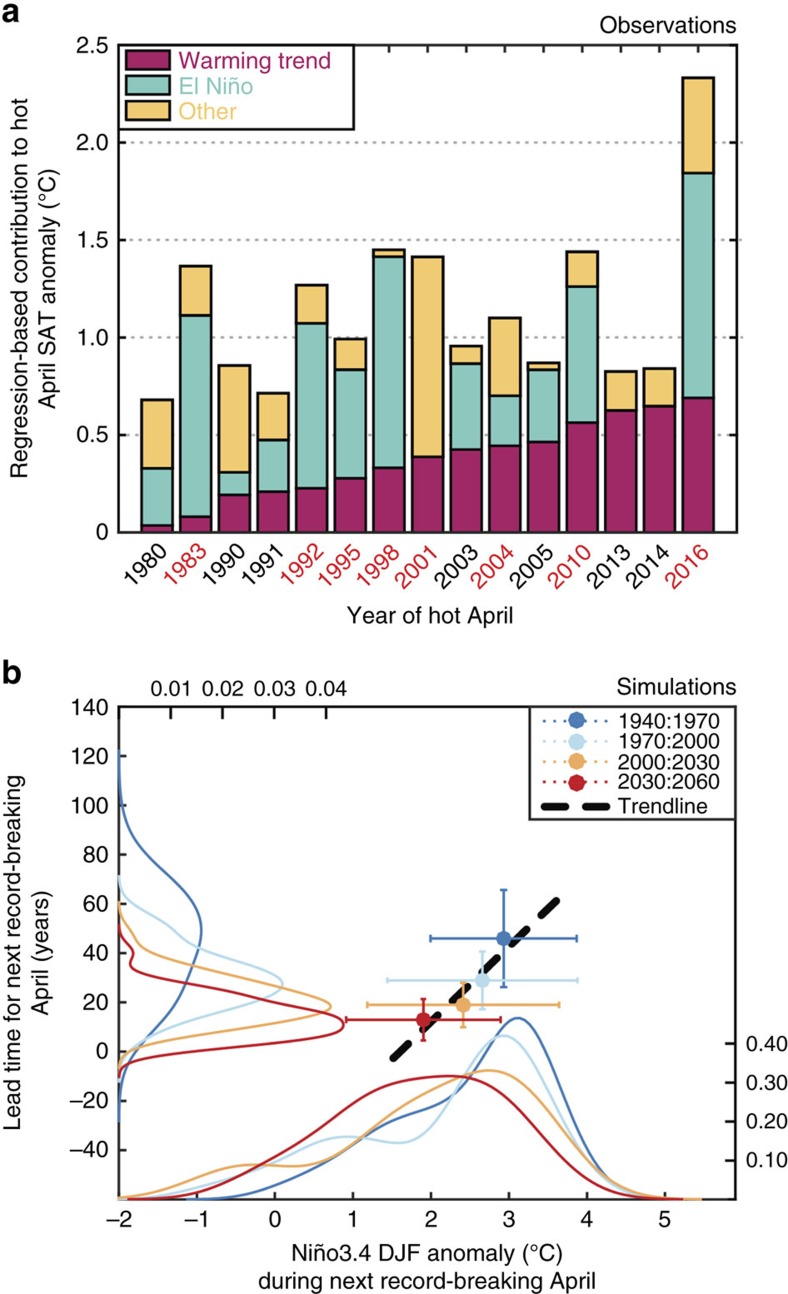
Attributing hot Aprils in Mainland Southeast Asia. (**a**) Observations: The relative contribution of El Niño (green bars) versus the long-term warming trend (red bars) towards the 15 hottest April SATs (>80th percentile) in the GISTEMP record of Mainland Southeast Asia (MSA; 1940–2016) using a regression model. The residual of the observed anomaly and the regression fit is termed as ‘other' variability (yellow bars). The years in red on the *x*-axis indicate the eight hottest extreme April events (>90th percentile). (**b**) Simulations: Median lead time for the next record-breaking April (years) compared to the baseline period (colours in legend) versus the median DJF Niño-3.4 SST anomaly (°C) for that forthcoming record-breaking year across all CESM1-LE members (with ±1*σ* ranges across the 39 members at each baseline period). A trendline for these datapoints calculated with the uncertainty is shown (black dashed line). Also depicted are the estimated probability density functions from the histograms of lead times as well as the DJF Niño-3.4 SST anomalies during the next record-breaking April at each baseline period.

We chose the 15 hottest Aprils (greater than the 80th percentile of the warmest Aprils) from the entire GISTEMP data set (1940–2016) and based on our regression model, investigated the influence of long-term warming versus El Niño in each April extreme (Fig. 4). All 15 events occur after 1980, i.e., in the latter, warmer subperiod of our previous analysis. April 2016 is the warmest on record, surpassing the previous record holder, April 1998, by 0.9 °C (Fig. 1c). Both of these record-breaking Aprils occurred after the peak of extremely strong El Niño events (1997–1998 and 2015–2016). The ENSO contribution typically accounts for 30–75% (0.25–1.15 °C) of the hot April SAT anomaly in MSA during those hot April events coinciding with El Niño years. Indeed, the eight hottest Aprils (>90th percentile) all coincided with El Niño years (labelled in red in Fig. 4a), with the notable exception of April 2001 where the residual term dominates. On the other hand, the recent hot Aprils of 2001, 2013 and 2014 do not occur during El Niño years, and as such, their anomalies (albeit minor) are mainly explained by long-term warming (Fig. 4). However, even though the regression model cannot fully explain the observed anomalies for these outliers (see residual component labelled ‘other' in Fig. 4a), it does indicate that long-term warming is increasingly playing a role (∼25–60%) in producing extreme April SATs in the absence of El Niño events. The regression model also indicates an upward trend in the relative contribution of long-term warming, which increases across these 15 events until 2016, and will foreseeably increase as regional warming continues to influence hot Aprils.

The regression model indicates that the long-term warming trend caused ∼29% of the extreme 2016 April SAT in MSA (Fig. 4a). Despite its unprecedented strength, El Niño accounted for ∼49% of the record-breaking hot April of 2016, leaving 22% unexplained. If some amount of the unexplained portion of the 2016 April SAT, apart from the contribution of weather-related noise, arose from a nonlinear change in the rate of global warming^30^, the imprint of regional warming on the 2016 extreme would be even larger than our estimate. Regardless of this unexplained portion, our regression model holds predictive value^18^ as the DJF Niño-3.4 SST anomaly can be observed months before the potential hot April.

### Future temperature extremes in MSA

With continued warming projected for the rest of this century^8^, will MSA experience more frequent, record-breaking hot Aprils? We investigated the likelihood of MSA extremes becoming more common in the future by analysing SAT data from the CESM1-LE. Starting from 1940 and using a non-overlapping moving window of 30 years, we calculated the hottest April in each of the ensemble members for that baseline period. For each of these record hot Aprils, we also calculated the DJF Niño-3.4 SST anomaly of that year. We then determined the lead time for the next, new, record-breaking April across each member (i.e. difference in years between the baseline record and new record). For each lead time, we also computed the DJF Niño-3.4 SST anomaly for that year in order to explore the control of El Niño on April extremes. Finally, we repeated this procedure for windows from 1940–1970 to 2030–2060 to understand the effect of warming on the lead-time for the next record-breaking April (Fig. 4b).

The median lead times across all ensemble members indicate that indeed, with future warming, record-breaking April SATs occur more frequently in MSA (Fig. 4b), a result in agreement with the statistics of record-breaking events^31^. The simulations suggest that record-breaking April SATs relative to an early twentieth century baseline occurred during large El Niño events (DJF Niño-3.4 SST anomaly >2 °C) whereas record-breaking April events in the twenty-first century can occur during El Niño events of smaller magnitudes. We note that 88.5% (414 out of 468) of all record-breaking extremes occur during El Niño years (DJF Niño-3.4 SST anomaly >0.5 °C), where 63% (260 out of 414) occur during strong events (DJF Niño-3.4 SST anomaly >2 °C). Thus, even though global warming will induce more extreme SATs in the future for the MSA region, our findings are advantageous for anticipating hot Aprils by monitoring the peak of Niño-3.4 SSTs a few months in advance.

## Discussion

Understanding and projecting the impacts of global warming on regional extremes is of critical importance for adaptation purposes^32^,^33^. On the spatiotemporal scales addressed in this study, the magnitude of natural variability is large relative to long-term trends and makes the detection and attribution of global warming more challenging. While located in the tropics, MSA is an exception to the general rule that anthropogenic warming will emerge sooner in the low latitudes, as shown by the prominent effect of ENSO on SAT variability in the region (Figs 1 and 2). The hot April of 2016 is an example of such ENSO-modulated extremes. Nevertheless, by focusing on post-El Niño Aprils (Fig. 3), and because the strong correlation with ENSO allows for the removal of its effect, we were able to detect the impact of long-term warming on observed record-breaking SATs in MSA (Fig. 4a). Despite a low number of El Niño events in the observational record, the CESM1-LE simulations provide conclusive statistics that long-term warming is increasingly aggravating the effect of El Niño in governing the frequency of these extremes (Fig. 4b). It is very likely that continued warming in the region will continue to conspire with El Niño in order to bring more common record-breaking extremes in the future. Even though the record-breaking April 2016 extreme in MSA was primed by the El Niño of 2015, compared to previous post-Niño extremes, the influence of long-term warming is incontrovertible. Furthermore, the influence of long-term warming on future El Niño impacts will only rise in importance. These extreme events could be quantitatively predicted some months in advance, and can thereby increase the preparedness of societies that will be impacted.

## Methods

### Surface temperature data sets

We used four different observational data sets for analysing surface temperature in MSA: GISTEMP^1^,^2^, CRU Ver 3.23 (ref. 3), HadISST1.1 (ref. 21) and the satellite-based MODIS data set^34^. The majority of our analysis on MSA were performed using the GISTEMP data set spanning from 1940 to 2016 (Figs 1, 2, 3, 4). 1940 was chosen as the start year due to the increasing density of temperature stations in MSA^1^,^2^, which is also reflected in the improved agreement between CRU and GISTEMP after 1940 (Supplementary Fig. 1). Composite post-Niño April analysis in Fig. 2c was performed jointly on the CRU (land) and HadISST (ocean) data sets from 1900 to 2014. We used the ICOADS^28^, ERA-Interim^27^ and HadSLP2 data sets^35^ to investigate cloud cover (Fig. 2b–d), surface energy fluxes (Supplementary Fig. 2) and sea-level pressure (Supplementary Fig. 3). We also use temperature station data for Bangkok (Supplementary Fig. 4) spanning from 1870 to 2013 taken from the Berkeley Earth Surface Temperature data set^36^. Though this was available from 1813, we only focus on the post-1870 period because of the availability of HadISST1.1 data to compute the Niño-3.4 SST index. Unless stated otherwise, we employed linear detrending to remove trends throughout our study. For both observations and simulations, we used the same spatial extent of the MSA box for analysis (6–22 °N, 94–110 °E).

### Climate model simulations

We used model output from the Community Earth System Model Version 1 (CESM1) which consists of a large ensemble (hereinafter CESM1-LE) of 39 simulation of climate from 1920 to 2100 (ref. 22). The large number of realizations allows us to perform a more robust statistical analysis of the link between El Niño and extreme April SATs for this study, as well attributes the changes to increasing anthropogenic forcings. All the simulations were run with historical forcings until the year 2005, after which the forcings follow the RCP8.5 scenario^8^. Each member is unique due to the application of a small, random perturbation to the initial air temperature at 1920, which leads to independent simulated trajectories in weather and internal climate variability among the members. However, since external forcings are the same, all the members contain the same anthropogenic (forced) response^22^.

ENSO variability arises spontaneously in the CESM1-LE set of simulations where many aspects of El Niño and La Nina events are realistically simulated, although El Niño events show amplitudes that exceed those of observed events^37^. For the composite analysis (Fig. 2c), El Niño events were selected from 1920 to 2015 in each member based on the peak of SST anomalies in the Niño-3.4 region that were larger than 0.5 standard deviations of the entire time series of each ensemble member. The composite plot was not sensitive to whether this threshold was increased to 0.75 standard deviations or 1 standard deviation. Over this time period, prior to picking El Niño events, we linearly detrended the Niño-3.4 SST timeseries simulated in each member to separate ENSO from the long-term warming trend. As stated in the main text, for plotting purposes, we downscaled the CESM1-LE composite SAT values by 0.45 over land and the oceans so that the April composite SST anomalies averaged over the Niño-3.4 region agree in magnitude with the observed April HadISST1.1 composite.

We also performed similar analyses on a 10-member ensemble of simulations from the Community Atmosphere Model Version 5 run in Tropical Ocean-Global Atmosphere configuration (CAM5-TOGA; Supplementary Fig. 5a). The CAM5-TOGA simulations, which are designed to simulate atmospheric variability uncoupled from the ocean, are run with observed SST fields in the tropics, whereby ENSO events occur with the same timing and amplitude as in nature. Thus, as opposed to the CESM1-LE, ENSO events are prescribed in these set of simulations. However, similar to the CESM1-LE, each member is uniquely different also due to small perturbations in initial air temperature conditions, resulting in independent weather trajectories. We find that composite analysis of post-Niño Aprils as well as histograms obtained from CAM5-TOGA agree with CESM1-LE and also with the observations (cf. Figs 2 and 3 and Supplementary Fig. 5).

Finally, we used the CESM1-LE set of simulations for analysing the lead time and the corresponding DJF Niño-3.4 SST anomaly for new record-breaking April SAT extremes relative to a baseline period (Fig. 4b). For this analysis, we used a non-overlapping sliding window from 1940 to 1970 until 2030 to 2060 and found that increasing/decreasing the baseline period by up to 10 years and/or allowing overlap did not change our findings, i.e., lead times and magnitude of DJF Niño3.4-SST anomalies required for future record-breaking Aprils decrease with the advent of long-term warming in MSA. We refrained from using the complete data set from 1920 to 2100 due to potential edge effects affecting our analysis (e.g. as detailed by Hawkins *et al*.^16^).

For the 1920–2060 time range, our ENSO definition is based on detrended Niño-3.4 data to separate ENSO from global warming. Instead of directly removing trends however, we removed the forced warming from the Niño3.4 timeseries, which we compute from the ensemble-mean Niño-3.4 timeseries. Small interannual variability remains in the ensemble mean, which we smooth using a 10-year low pass filter. Then we remove this estimate of the forced warming from the Niño-3.4 index from each individual member to isolate the unforced variability. We chose El Niño (La Niña) events as those years where peak SSTs were higher (lower) than 0.5 standard deviations of the overall timeseries in each member after subtracting the 10-year smoothed ensemble mean of Niño-3.4. Our results were not affected if we chose 0.5 standard deviations or 1 standard deviation to pick ENSO events. The trend line in Fig. 4b (black dashed line) was calculated using a maximum likelihood regression methodology that incorporates bivariate error^38^,^39^.

### Multivariate regression

We regressed April SATs in MSA upon linear and quadratic components of the long-term warming trend along with a term proportional to the DJF Niño-3.4 SST Index and a residual term in the form *SAT*_April_=*αt*+*βt*^2^+*γT*+*δ* where *t* is time in years, *T* is the DJF Niño-3.4 SST anomaly and *α*,*β*, and *γ* are the regression coefficients. The fit yielded a high correlation coefficient (*r*=0.83; Supplementary Fig. 6), which indicates that ENSO and global warming explain much of the April SAT variability. The analysis and results were similar even when we used only the December Niño-3.4 SST anomaly (*r*=0.76), no quadratic long-term warming term and only a linear component (*r*=0.74), or global mean surface temperatures instead of April SATs in MSA (*r*=0.80). However, the regression configuration used in the main text provided the highest correlation coefficient. We used this regression to attribute April extremes in MSA to El Niño and long-term warming (Fig. 4a). The negative contributions of El Niño, although minimal (<10%) for the three non-El-Niño extremes (2001, 2013, and 2014), are not shown. The ‘other' variability was calculated as the difference between the observed anomaly and the residual fit (where the negative El Niño contributions were set to zero where they applied).

### Code availability

The MATLAB codes that have contributed to the results and analysis reported in this study are readily available upon request from the lead author (K.T.: kau@ig.utexas.edu; Git: holy-kau).

### Data availability

The observational/reanalysis data sets used in this study are available here: the GISTEMP data set^2^— https://data.giss.nasa.gov/gistemp/, the CRU Ver 3.23 data set^3^— https://crudata.uea.ac.uk/cru/data/hrg/cru_ts_3.23/, the HadISST1.1 data set^21^— http://www.metoffice.gov.uk/hadobs/hadisst/, the MODIS data set^34^— https://lpdaac.usgs.gov/dataset_discovery/modis, the ERA-Interim data set^27^— https://esrl.noaa.gov/psd/data/gridded/data.erainterim.html, the ICOADS data set^28^— http://icoads.noaa.gov, the HadSLP2 data set^35^— http://www.metoffice.gov.uk/hadobs/hadslp2/, the BEST data set^36^— http://berkeleyearth.org. Output from the CESM large ensemble^22^ simulations can be found at http://www.cesm.ucar.edu/projects/community-projects/LENS/ and the CMIP5 (ref. 23) simulations at https://esgf-node.llnl.gov/.

## Additional information

**How to cite this article:** Thirumalai, K. *et al*. Extreme temperatures in Southeast Asia caused by El Niño and worsened by global warming. *Nat. Commun.* **8**, 15531. doi: 10.1038/ncomms15531 (2017).

**Publisher's note**: Springer Nature remains neutral with regard to jurisdictional claims in published maps and institutional affiliations.

## Supplementary Material

Supplementary Information Supplementary Figures, Supplementary Discussion and Supplementary References

## References

[b1] Hansen J., Ruedy R., Sato M. & Lo K. Global surface temperature change. Rev. Geophys. 48, RG4004 (2010).

[b2] GISTEMP Team and others. *Giss Surface Temperature Analysis (gistemp)* (NASA Goddard Institute for Space Studies, 2016). Retrieved 5/1/2016 from http://data.giss.nasa.gov/gistemp/.

[b3] Harris I., Jones P. D., Osborn T. J. & Lister D. H. Updated high-resolution grids of monthly climatic observations—the CRU TS3.10 Dataset. Int. J. Climatol. 34, 623–642 (2013).

[b4] Electricity Generating Authority of Thailand. Electricity peak demand hits 28,351.7 MW, breaking the peak record for the second time in 2016. http://www.egat.co.th/en/index.php?option=com_content&view=article&id=326:electricity-peak-demand-hits-28-351-7-mw-breaking-the-peak-record-for-the-catid=11&Itemid=112 (2016).

[b5] Associated Press. Scorching heat wave in Thailand is longest in 65 years. http://www.chicagotribune.com/news/nationworld/ct-thailand-hot-april-20160427-story.html (2016).

[b6] Nirmal Ghosh. Heatwave In Asia: coffee crops die in Vietnam, Thai rice yield shrinks. http://www.asianews.network/content/heatwave-asia-coffee-crops-die-vietnam-thai-rice-yield-shrinks-15252 (2016).

[b7] Singhrattna N., Rajagopalan B. & Kumar K. K. Interannual and interdecadal variability of Thailand summer monsoon season. J. Climate 18, 1697–1708 (2005).

[b8] Stocker T. F. . Summary for policymakers. Climate Change 2013: the Physical Science Basis Contribution of Working Group I to the Fifth Assessment Report of the Intergovernmental Panel on Climate Change Cambridge University Press (2013).

[b9] Manton M. J. . Trends in extreme daily rainfall and temperature in Southeast Asia and the South Pacific: 1961–1998. Int. J. Climatol. 21, 269–284 (2001).

[b10] Mahlstein I., Knutti R., Solomon S. & Portmann R. W. Early onset of significant local warming in low latitude countries. Environ. Res. Lett. 6, 034009 (2011).

[b11] Diffenbaugh N. S. & Scherer M. Observational and model evidence of global emergence of permanent, unprecedented heat in the 20th and 21st centuries. Climatic Change 107, 615–624 (2011). 22707810 10.1007/s10584-011-0112-y PMC3374649

[b12] Hawkins E. & Sutton R. Time of emergence of climate signals. Geophys. Res. Lett. 39, L01702 (2012).

[b13] Lehner F., Deser C. & Sanderson B. M. Future risk of record-breaking summer temperatures and its mitigation. Climatic Change doi: 10.1007/s10584-016-1616-2 (2016).

[b14] Herring S. C., Hoerling M. P. & Kossin J. P. Explaining extreme events of 2014 from a climate perspective. Bull. Am. Meteorol. Soc. 96, S1–S172 (2015).

[b15] Mahlstein I., Hegerl G. & Solomon S. Emerging local warming signals in observational data. Geophys. Res. Lett. 39, L21711 (2012).

[b16] Hawkins E. . Uncertainties in the timing of unprecedented climates. Nature 511, E3–E5 (2014). 24990757 10.1038/nature13523

[b17] King A. D. . The timing of anthropogenic emergence in simulated climate extremes. Environ. Res. Lett. 10, 094015 (2015).

[b18] Nicholls N. . The El Niño–Southern oscillation and daily temperature extremes in east Asia and the west Pacific. Geophys. Res. Lett. 32, L16714–L16714 (2005).

[b19] Trenberth K. E. The definition of El Niño. Bull. Am. Meteorol. Soc. 78, 2771–2777 (1997).

[b20] Trenberth K. E., Caron J. M., Stepaniak D. P. & Worley S. Evolution of El Niño Southern Oscillation and global atmospheric surface temperatures. J. Geophys. Res. 107, AAC 5–1–AAC 5–17 (2002).

[b21] Rayner N. A. . Global analyses of sea surface temperature, sea ice, and night marine air temperature since the late nineteenth century. J. Geophys. Res. 108, 4407–4437 (2003).

[b22] Kay J. E. . The Community Earth System Model (CESM) Large Ensemble Project: a community resource for studying climate change in the presence of internal climate variability. Bull. Am. Meteorol. Soc. 96, 1333–1349 (2015).

[b23] Taylor K. E., Stouffer R. J. & Meehl G. A. An overview of CMIP5 and the experiment design. Bull. Am. Meteorol. Soc. 93, 485–498 (2012).

[b24] Halpert M. S. & Ropelewski C. F. Surface temperature patterns associated with the Southern Oscillation. J. Climate 5, 577–593 (1992).

[b25] Trenberth K. E. & Fasullo J. T. Climate extremes and climate change: the Russian heat wave and other climate extremes of 2010. J. Geophys. Res. 117, D17103 (2012).

[b26] Klein S. A., Soden B. J. & Lau N.-C. Remote sea surface temperature variations during ENSO: evidence for a tropical atmospheric bridge. J. Climate 12, 917–932 (1999).

[b27] Dee D. P. . The ERA-Interim reanalysis: configuration and performance of the data assimilation system. Q. J. R. Meteorol. Soc. 137, 553–597 (2011).

[b28] Worley S. J., Woodruff S. D., Reynolds R. W., Lubker S. J. & Lott N. ICOADS release 2.1 data and products. Int. J. Climatol. 25, 823–842 (2005).

[b29] Meehl G. A. & Tebaldi C. More intense, more frequent, and longer lasting heat waves in the 21st century. Science 305, 994–997 (2004). 15310900 10.1126/science.1098704

[b30] Meehl G. A., Teng H. & Arblaster J. M. Climate model simulations of the observed early-2000s hiatus of global warming. Nat. Climate Change 4, 898–902 (2014).

[b31] Benestad R. E. Record-values, nonstationarity tests and extreme value distributions. Global Planet. Change 44, 11–26 (2004).

[b32] Fedoroff N. V. . Radically rethinking agriculture for the 21st century. Science 327, 833–834 (2010). 20150494 10.1126/science.1186834 PMC3137512

[b33] Luo J.-J. . Impacts of El Niño Southern Oscillation on the global yields of major crops. Nat. Commun. 5, 1–7 (2014). 10.1038/ncomms4712 24827075

[b34] Wan Z., Zhang Y., Zhang Q. & Li Z. Validation of the land-surface temperature products retrieved from Terra Moderate Resolution Imaging Spectroradiometer data. Remote Sensing Environ. 83, 163–180 (2002).

[b35] Allan R. & Ansell T. A new globally complete monthly historical gridded mean sea level pressure dataset (HadSLP2): 1850–2004. J. Climate 19, 5816–5842 (2006).

[b36] Rohde R. . Berkeley earth temperature averaging process. Geoinformatics & Geostatistics: an Overview 1,, doi: 10.4172/2327-4581.1000103 (2013).

[b37] DiNezio P. N., Barbero L., Long M. C. & Lovenduski N. Are anthropogenic changes in the tropical ocean carbon cycle masked by Pacific Decadal Variability? US CLIVAR Variations 13, 12–23 (2015).

[b38] Thirumalai K., Singh A. & Ramesh R. A MATLAB code to perform weighted linear regression with (correlated or uncorrelated) errors in bivariate data. J. Geol. Soc. India 77, 377–380 (2011).

[b39] York D., Evensen N. M., Martnez M. L. & De Basabe Delgado J. Unified equations for the slope, intercept, and standard errors of the best straight line. Am. J. Phys. 72, 367–369 (2004).

