# Supplementary material for: Extreme temperatures in Southeast Asia caused by El Niño and worsened by global warming
Source: Nat Commun. 2017 Jun 6;8:15531. doi: 10.1038/ncomms15531 (PMC5467164; doi:10.1038/ncomms15531)
Supplement: Supplementary Information — Supplementary Figures, Supplementary Discussion and Supplementary References [file ncomms15531-s1.pdf]

Supplementary Discussion

The Link between El Niño and MSA April SATs: Our study finds a robust relationship between ENSO and April SAT in MSA. This link is critical for our regression analysis where ENSO and long-term warming components explain the observed SAT anomalies with a high correlation coefficient (r=0.83; Supplementary Fig. 6). Moreover, according to the reanalysis data (Fig. 2b), the reductions in cloud cover are most pronounced over MSA and over the Philippines. We explored the composite evolution of anomalous SAT, cloud, rainfall, and surface heat fluxes averaged over MSA (Supplementary Fig. 2). This analysis allows us to explore how these variables evolve from the peak of El Niño events (time = 0) to the April peak warming (time = 4) and its decay thereafter.

Both reanalysis and CESM1 data show negligible MSA warming at the peak of El Niño relative to its peak around March and April (Supplementary Fig. 2). Cloud cover and rainfall progressively decrease following the warming of MSA reaching a peak around March-April (Supplementary Fig. 2). Associated with these changes, there is an increase in surface radiation and latent heat flux (positive downwards) balanced by a decrease in sensible heat flux (Supplementary Fig. 2). Both ERA-Interim as well as the CESM1-LE simulations indicate reductions in clear-sky radiation and diminished sensible heat fluxes during post-Niño Aprils with increased surface energy and latent heat fluxes due to diminished cloud coverage (Supplementary Fig. 2).

The progressive increase in these quantities could be explained by a positive land-atmosphere feedback amplifying the initial warming driven by El Niño. The initial warming following the peak of El Niño could reduce cloud cover, which further amplifies the warming. The increase in latent head flux indicates reduced evaporative cooling associated with land drying, which would also act to amplify the warming. This positive feedback is more evident in the CESM1-LE than the ERA reanalysis, and could explain the enhanced and delayed warming over MSA and the fact that this region exhibits the strongest correlation with peak El Niño amplitude throughout the global

tropics. Conversely, it is possible that the enhanced April warming is driven by the circulation anomalies associated with El Niño, particularly the subsiding branch, which appears to migrate from the Maritime Continent to the MSA and the Philippines following the seasonal migration of the monsoon circulation.

Correlation patterns of temperature and sea-level pressure indicate a potential link with the tropical Indian Ocean (Supplementary Fig. 3). As previous studies have suggested[1,2], the Indian Ocean might play a role as a "capacitor" in remaining warm after an El Niño event begins to decay. Subsequently, this triggers anomalous anticyclones in the Indo-western Pacific region which can suppress convection. This can delay the onset of monsoonal rainfall and clouds and hence exacerbate warming over MSA[2]. Although these studies indicate that this effect is maximal during the July-August season, this "capacitor effect" might explain some of the relationship between April SATs in MSA and El Niño. The residual correlation patterns of SAT and SLP that emerge after removing the regressed component of Niño-3.4 SSTs display this link with the Indian Ocean but also indicate that internal atmospheric variability over Eurasia may also affect MSA SAT variability (Supplementary Fig. 3c-d). These large-scale patterns might also explain some of the observed intra-extreme event variability (Fig. 3a and Supplementary Figs. 4-5).

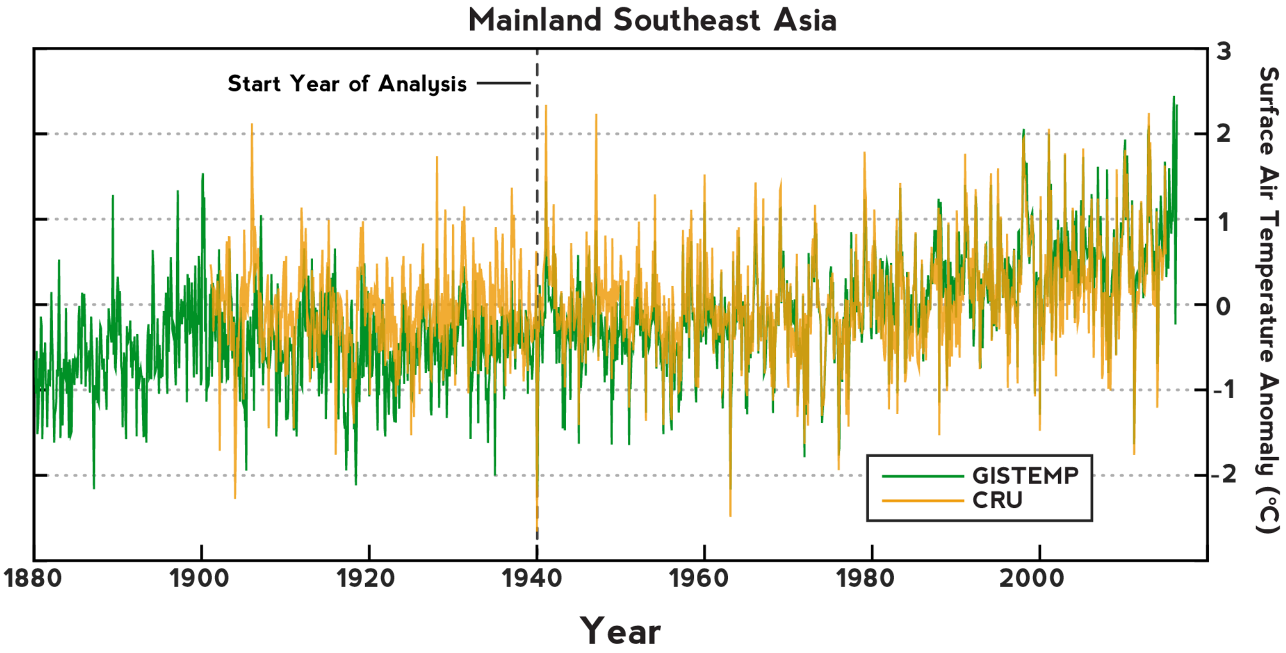

Supplementary Figure 1. Mainland Southeast Asia Surface Air Temperature Record Monthly-mean surface air temperature (SAT) anomalies for the Mainland Southeast Asia (MSA) region (6-22°N, 94-110°E) from the CRU (yellow; 1900-2014) and GISTEMP (green; 1880-2016) datasets. Black dashed line at 1940 indicates the start year chosen for analysis from GISTEMP, when temperature station density increased in MSA, and when both datasets align.

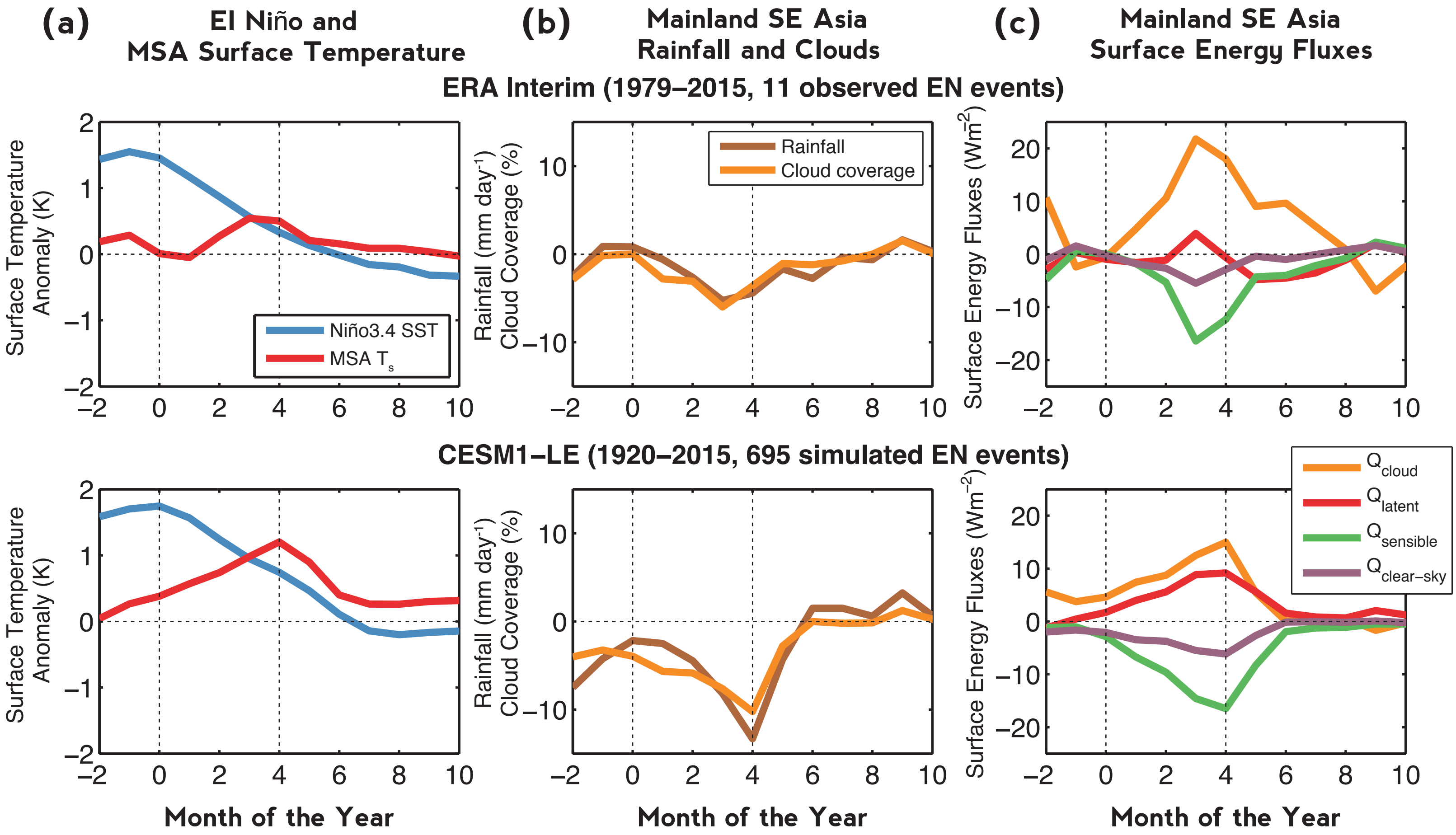

Supplementary Figure 2. Simulated evolution of MSA climate anomalies during the peak and decay phase of El Niño (a) Composite Niño-3.4 SST index (blue line) and MSA surface temperature anomalies in K (red line). (b) Composite of MSA rainfall in mm day^{-1} (brown line) and cloud coverage in % (orange line). (c) Composite of surface energy budget terms in W m^{-2}: surface radiation due to clouds (orange line), latent heat flux (red line), clear-sky radiation (purple) and sensible heat flux (green). Time (x-axis) is given in months after the peak of El Niño in January (month = 0). The evolution of all composite variables is shown from November prior to the peak of El Niño (month = -2) to Novemeber of the subsequent year (month = 10).

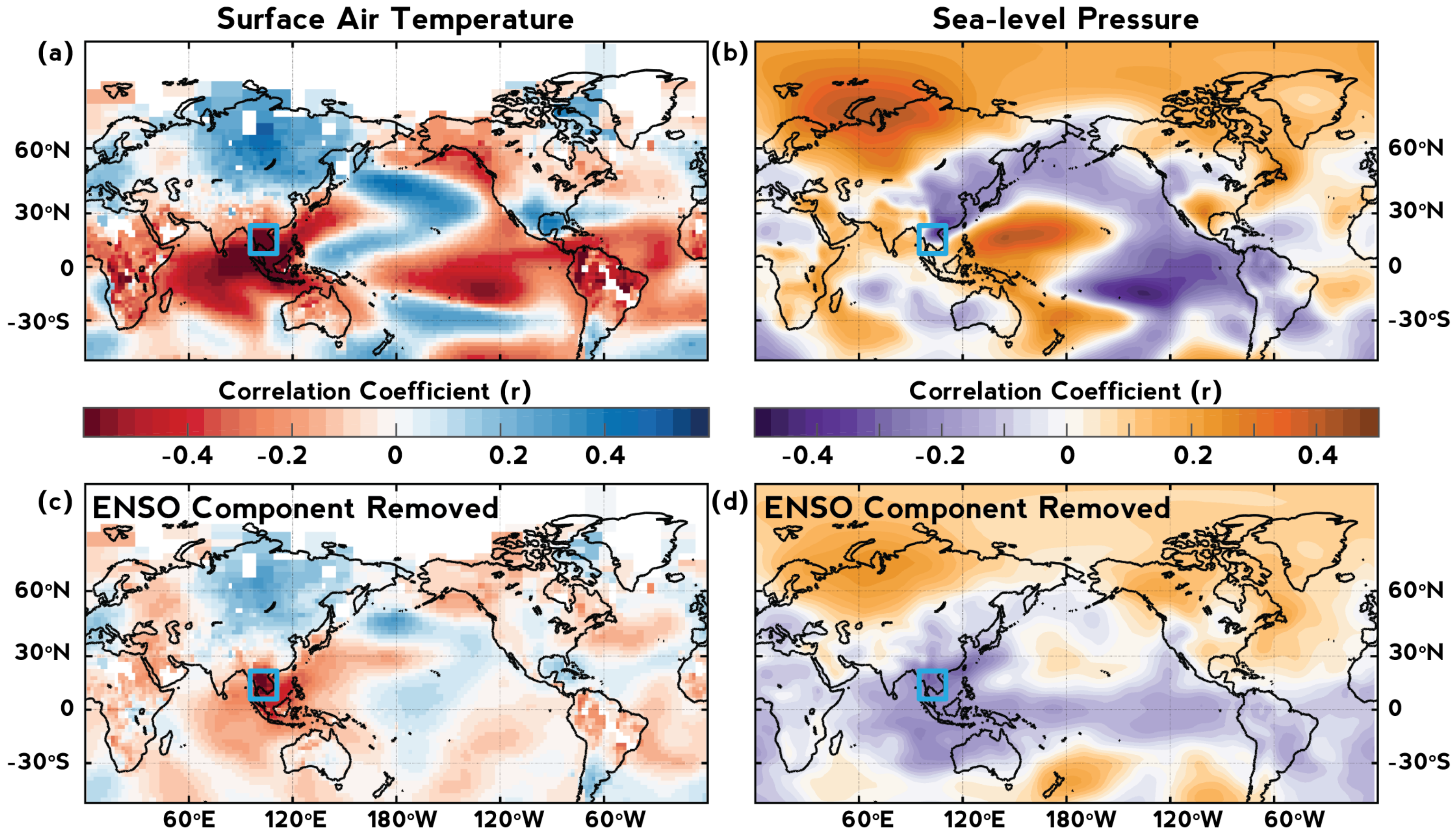

Supplementary Figure 3. Correlations of MSA surface air temperature during April Above: Correlation between April SAT in MSA (blue box) with global SAT (a) and global sea-level pressure (b; SLP) during April. Below: Correlation between residual April SAT in MSA (blue box) and global SAT (c) and SLP (d) after the regression of DJF Niño-3.4 SSTs upon the MSA April values were removed.

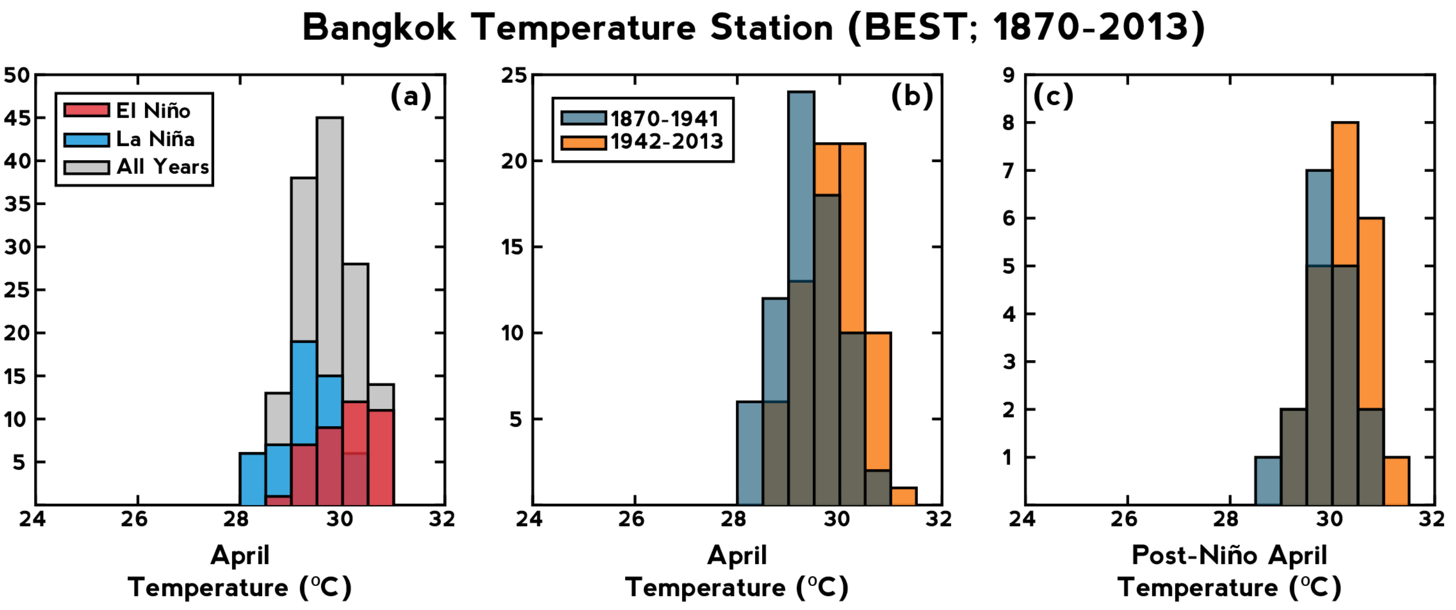

Supplementary Figure 4. Temperature station data from Bangkok Histograms of (a) all Aprils divided into El Niño, La Niña, and all years from the linearly detrended Bangkok temperature station data (1870-2013), (b) all April data divided into two equal subperiods (dark blue - 1870-1941; orange - 1942-2013), and (c) only post-Niño Aprils divided into equal subperiods in the Bangkok timeseries. Data in each subperiod was linearly detrended prior to analysis. Data were obtained from the Berkeley Earth Surface Temperature (BEST) database

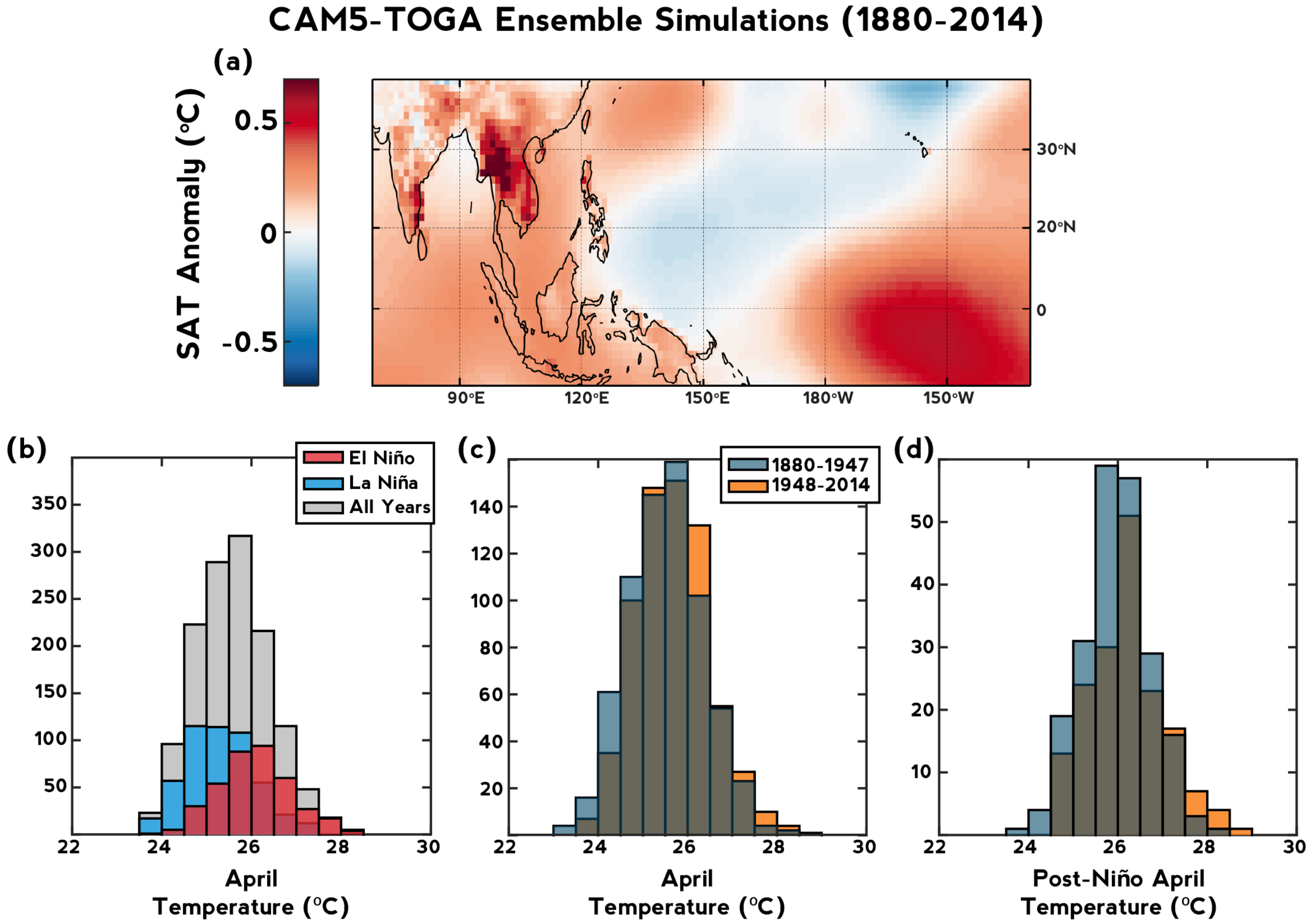

Supplementary Figure 5. Atmosphere-Only Ensemble Simulations Above: (a) Composite SAT of Aprils after the peak of El Niño events across all members from the CAM5-TOGA ensemble (n = 10). Below: Histograms of (b) All Aprils divided into El Niño, La Niña, and all years from the linearly detrended CAM5-TOGA ensemble members (1880-2014), (c) all April data divided into two equal subperiods (dark blue - 1880-1947; orange - 1948-2014), and (d) only post-Niño Aprils in the MSA timeseries. Data in each subperiod was linearly detrended prior to histogram analysis.

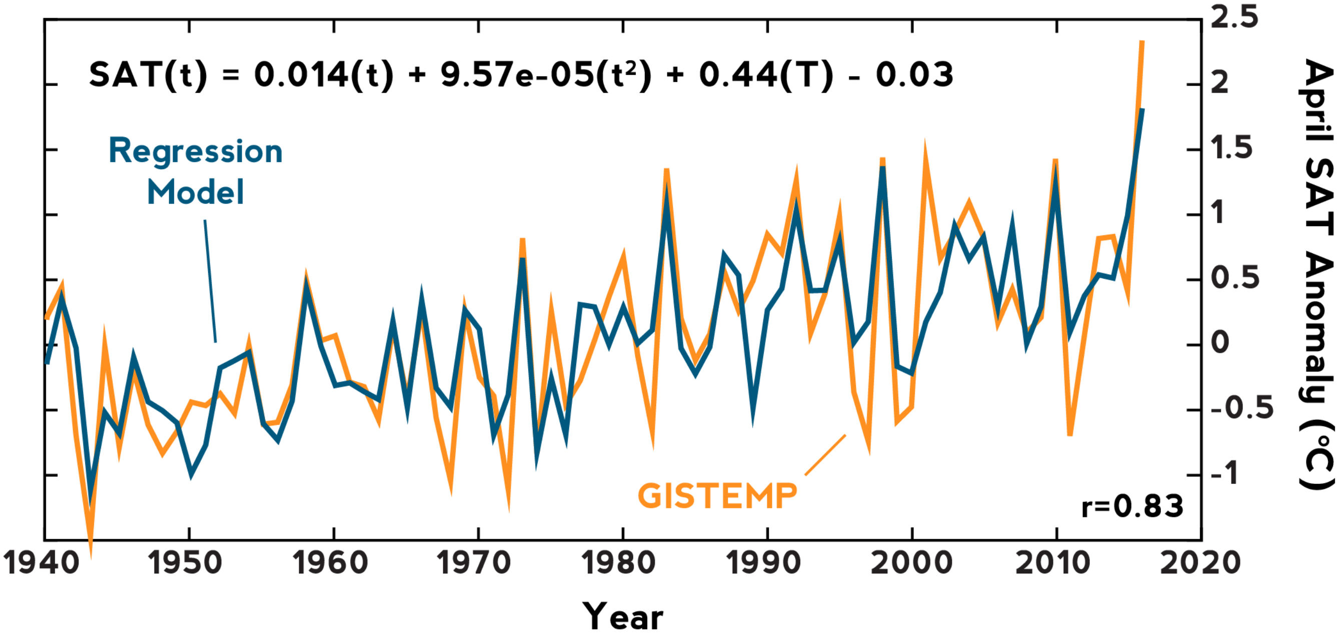

Supplementary Figure 6. Regression model for April SATs The fit obtained from regressing ENSO variability and the long-term warming trend in MSA onto April SAT anomalies (blue) versus observed April SAT anomalies from the GISTEMP dataset (orange). The equation with regression coefficients is displayed at the top where 't' indicates the year of April SAT, and 'T' indicates the DJF Niño-3.4 SST anomaly. Note that the correlation coefficient between the two curves is 0.83.

Supplementary References

1. Xie, S.-P. *et al.* Decadal Shift in El Niño Influences on Indo–Western Pacific and East Asian Climate in the 1970s*. *Journal of Climate* **23**, 3352–3368 (2010). URL http://journals.ametsoc.org/doi/abs/10.1175/2010JCLI3429.1.

2. Xie, S.-P. *et al.* Indian Ocean Capacitor Effect on Indo–Western Pacific Climate during the Summer following El Niño. *Journal of Climate* **22**, 730–747 (2009). URL http://journals.ametsoc.org/doi/abs/10.1175/2008JCLI2544.1.
